# Immune correlates of CD4 decline in HIV-infected patients experiencing virologic failure before undergoing treatment interruption

**DOI:** 10.1186/1471-2334-8-59

**Published:** 2008-05-02

**Authors:** Kenneth H Huang, Mona R Loutfy, Christos M Tsoukas, Nicole F Bernard

**Affiliations:** 1 Research Institute of the McGill University Health Center, Montreal, Quebec, Canada; 2 University of Toronto, Toronto, Ontario, Canada

## Abstract

**Background:**

The advantage of treatment interruptions (TIs) in salvage therapy remains controversial. Regardless, characterizations of the correlates of CD4 count fall during TI are important to identify since patients with virologic failure commonly stop antiretroviral (ARV) therapy. The objective of this study was to determine the predictive value of pre-TI proliferative capacity and cell surface markers for CD4 count change in HIV-infected patients experiencing virologic failure before undergoing TI.

**Methods:**

Peripheral blood mononuclear cells (PBMCs) from 13 HIV-infected patients experiencing virologic failure at baseline time points before the TI were tested for proliferation using the 5,6-carboxyfluorescein diacetate succinimidyl ester (CFSE) dilution assay and a Gag p55 peptide pool, staphylococcus enterotoxin B (SEB), cytomegalovirus (CMV) recall antigen, and anti-CD3 antibody as stimuli. CD28 and CD57 expression on CD4+ and CD8+ T-cells was measured.

**Results:**

The median changes in the CD4+ T-cell count and viral load from baseline to the TI time point corresponding to the CD4 count nadir were -44 cells/mm^3 ^{Interquartile range (IQR) -17, -104} and +85,332 copies/mL (IQR +11,198, +283,327), respectively. CD4+ T-cell proliferation to CMV, pre-TI CD4+ T-cell count, and percent CD4+CD57+ cells correlated negatively with CD4 count change during TI (r = -0.59, p = 0.045, r = -0.61, p = 0.030 and r = -0.69, p = 0.0095, respectively; Spearman correlation). The presence of HIV-specific proliferative responses was not associated with a reduced decline in CD4 count during TI.

**Conclusion:**

The use of pre-TI immune proliferative responses and cell surface markers may have predictive value for CD4 count decline during TI.

## Background

Combination antiretroviral (ARV) therapy (cART) has revolutionized the treatment of human immunodeficiency virus (HIV) infection, which is now viewed as a chronic and manageable disease with reduced rates of acquired immunodeficiency syndrome (AIDS)-related events and deaths [1]. However, the management of patients with multi-drug resistant HIV remains challenging, requiring the development of both new ARVs and novel treatment strategies to optimize outcomes in these patients.

One strategy that has been assessed in the context of treating multi-drug resistant HIV is the use of a structured treatment interruption (TI) [2-4]. In this setting, patients remain off ARVs for an often pre-determined duration of time prior to starting a new treatment regimen. The rationale for TI is based on the hypothesis that withdrawal of drug pressure may allow the re-emergence of wild-type virus, which would be more susceptible to suppression upon starting a new treatment regimen. In addition, TI may be used to alleviate existing side-effects and re-motivate the patient to adhere to subsequent therapy [2-4].

Despite the theoretical advantages, several concerns remain regarding the use of TI as a therapeutic strategy. Specifically, the eventual re-emergence of resistant virus from latently infected cells upon restarting ARV may lead to a rebound in viremia and an accompanying significant fall in CD4 T-cell count [4]. The most rapid decline in CD4 count is associated with the emergence of wild-type virus and increased viral replication [2]. The fall in CD4 count potentially places patients at increased risk of developing opportunistic infections, as was observed in the SMART trial, which investigated TIs as a therapeutic strategy [4,5]. Therefore, TIs are currently not recommended as a treatment strategy on a routine basis [4,5]. However, it is worthwhile to characterize immune correlates of CD4 decline during a TI as patients may elect to stop ARV therapy for various reasons, including virologic failure, pill fatigue and excessive toxicity.

In this study, we examined several immunological parameters observed at baseline time points within 12 months of TI to assess whether they were associated with the extent of CD4 count change during TI. The immunological parameters studied, which included the expression of CD28 and CD57 on T-cells, as well as T-cell proliferative potential, were selected because there existed a rationale for their association with CD4 count loss and viral load outcome during TI. The loss of CD28 expression on CD8 T-cells has been shown to correlate with HIV disease progression and loss of IL-2 production for autocrine proliferation [6,7]. The expression of CD57 identifies replicative senescence in HIV-specific T-cells, which are unable to proliferate and have a history of increased cell divisions [8,9]. HIV-specific proliferative potential is maintained in HIV infected individuals with favourable clinical outcomes such as long-term non-progessors and successfully treated aviremic patients [10,11]. The objective of this retrospective study was to evaluate the correlation between baseline T-cell proliferative capabilities and phenotypic markers with CD4 count decline during a TI in HIV-infected patients experiencing virologic failure.

## Methods

### Study Design

A retrospective study was carried out on cryopreserved peripheral blood mononuclear cell (PBMC) samples from 13 HIV-infected patients experiencing virologic failure on cART before undergoing a TI. PBMC from a single time point within 12 months before the TI were used as the baseline samples. All were receiving cART consisting of at least 3-drugs prior to the TI, which lasted at least 2 months. Virologic failure was defined as having plasma HIV-1 RNA level >5,000 copies/mL while taking cART measured on 2 occasions at least 4 weeks apart. The patients were followed at the Immune Deficiency Treatment Centre of the McGill University Health Centre in Montreal, Quebec, Canada. The study population included all the individuals identified at this clinical site who met the inclusion criteria and for whom frozen cell samples were available using a database that is maintained in our centre where clinical test results are collected at each clinic visit.

### Proliferation assay and flow cytometry analysis of CD28 and CD57 expression

Proliferative capacity was measured using the 5,6-carboxyfluorescein diacetate succinimidyl ester (CFSE, Molecular Probes) dilution assay. PBMCs were labeled with CFSE at a final concentration of 5 μM and stimulated with a pool of 122 HIV 15-mer peptides with 11 amino acid overlaps corresponding to the HIV-1 HXB2 Gag p55 sequence (NIH AIDS Research and Reference Reagent Program), cytomegalovirus (CMV) lysate (Advanced Biotechnologies Inc.), staphylococcus enterotoxin B (SEB) (Sigma-Aldrich), and anti-CD3 (Research Diagnostics) for 6 days at 37°C. On day 6, samples were stained with phycoerythrin (PE) conjugated anti-CD4, allophycocyanin (APC) conjugated anti-CD8, and peridinin chlorophyll protein (PerCP) conjugated anti-CD3 (BD Biosciences). The percent of CFSE^lo ^CD3+CD4+ and CD3+CD8+ T cells was analyzed using a FACSCalibur instrument (BD Biosciences) and FlowJo software (version 5.72; TreeStar). Anti-CD28-PE and anti-CD57 conjugated fluorescein isothiocyanate (FITC) were used to detect the percent of CD4+ and CD8+ T-cells expressing CD28 and CD57.

### Statistical analysis

Statistical analyses were performed using GraphPad InStat software (version 3.06; GraphPad). The significance of correlations between percent CFSE^lo ^cells generated to each stimulus, the percent of CD28+ and CD57+ T-cells and the pre-TI CD4 count with changes in the absolute number of CD4+ T-cells from the pre-TI time point to the CD4 count nadir (CD4+ T-cell decline) during the TI were assessed using the Spearman nonparametric correlation test. In addition, CD4+ T-cell change during TI was dichotomized based on the median into those with greater versus lesser CD4 count loss. These subpoplulations were compared for median percent CFSE^lo ^cells and the median percent of CD28+ and CD57+ expression on CD4+ and CD8+ T-cells using the Mann-Whitney test. All tests for statistical significance were two-tailed and *p *values <0.05 were considered significant.

## Results

### Description of study population

The study subjects had a median age of 42 years (IQR, 40, 43) and 11 (85%) were male and 2 (15%) were female. The median duration on ARV therapy was 8.5 years (IQR, 5.7, 10.7). The median number of previous ARV regimens and number of ARV drugs being taken at baseline were 12 (IQR, 8, 16) and 4.0 (IQR 3, 5), respectively. Prior to beginning a TI, 23% of patients were on a non-nucleoside reverse transcriptase inhibitor (NNRTI)-based regimen, 46% on a protease inhibitor (PI)-based regimen, 23% were on a regimen that included both PI- and NNRTI, and 8% were on a PI- and fusion inhibitor-based regimen. The median baseline viral load, baseline CD4 count and CD4 count nadir during TI were 24,719 copies/mL (IQR, 12,581, 161,918), 171 cells/mm^3 ^(IQR, 56, 264), and 28 cells/mm^3 ^(IQR, 9, 72), respectively. The median CD4 count decline (baseline to nadir) during TI was -44 cells/mm^3 ^(IQR, -17, -104). The median time from the on-therapy baseline CD4 count to the TI was 4.4 months (IQR, 2.3, 6.0) and from the on-therapy baseline CD4 count to the CD4 count nadir during the TI was 5.7 months (IQR, 3.1, 7.5).

### Baseline immune parameters and their association with CD4 count change during TI

**Figure 1:**
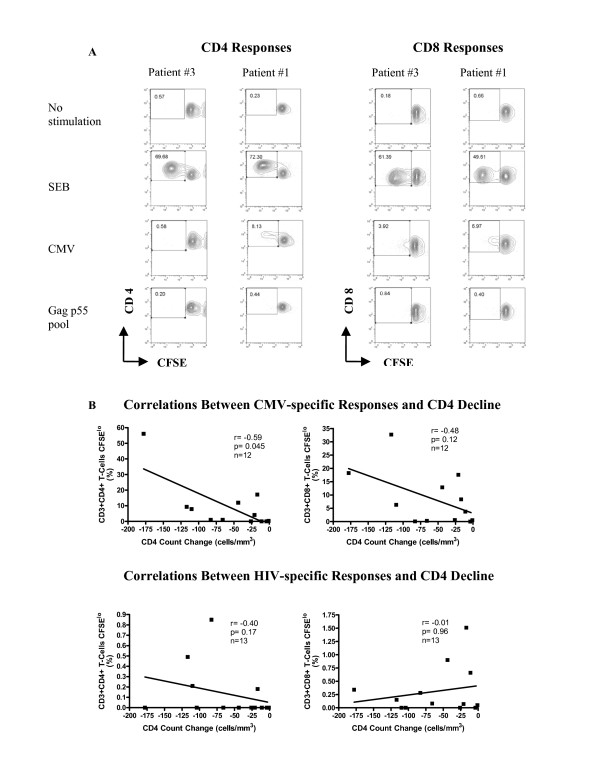
**Panel A shows representative FACS contour plots gated on CD3+ lymphocytes showing CD4+ and CD8+ proliferative responses to no stimulation (negative control), SEB (superantigen), CMV (recall antigen) and Gag p55 peptide pool (HIV-specific antigens) for a patient with a CD4 count change during TI that was lower than the median for the group (Patient #3) in the left hand panels and a patient with a change in CD4 count during TI that was higher than the median for the group (Patient #1) in the right hand panels.** The number in the box of each contour plot is the percentage of CFSE^lo ^cells, which is an indication of the level of proliferation that has occurred in response to that stimulus. In **Panel B**, results from all patients are plotted for which proliferation data was available. The percentage of CFSE^lo ^cells following stimulation is plotted on the y-axis and CD4 count change from baseline to nadir during TI on the x-axis. The left hand panels show the percentage of CFSE^lo ^cells in the CD4+ T cell compartment and the right hand panels the percentage of CFSE^lo ^cells in the CD8+ T cell compartment. The upper panels show proliferation to CMV lysate (n = 12) and the bottom panels to the HIV Gag p55 peptide pool (n = 13). Proliferation to CMV and the Gag p55 peptide pool was assessed on baseline time points. The line in each graph represents a trendline through the data points. Spearman nonparametric correlation tests were used to test the significance of the association between these parameters; and *p *values <0.05 were considered significant.

Figure 1A shows flow cytometry contour plots generated by measuring proliferation using a CFSE dilution assay in PBMCs from 2 study patients, one who experienced a CD4 count change during TI that was lower and the other higher than the median for the study population tested. The proliferative capacity of patient PBMC was screened using a panel of stimuli including no stimulation as a negative control, SEB and anti-CD3 antibody as positive controls, CMV lysate as a recall antigen and a Gag p55 peptide pool as an HIV-specific antigen. Figure 1B shows data for 12 patients stimulated with CMV lysate and 13 patients stimulated with the HIV Gag p55 peptide pool. One patient could not be tested for proliferation to CMV lysate due to an insufficient number of cells available. When the percentage of CFSE^lo ^T-cells was plotted versus CD4 count change from baseline to CD4 nadir during TI time points we found a statistically significant negative correlation between the percentage of CMV-specific CFSE^lo ^CD3+CD4+ T-cells and CD4 count change (r = -0.59, p = 0.045; Spearman correlation test). We also noted a negative correlation between pre-TI CD4 count and CD4 count change (r = -0.61, p = 0.030, Table 1). No significant relationship was observed between CMV-specific proliferation by CD8+ T-cells nor HIV Gag p55-specific proliferation by either CD4+ or CD8+ T-cells with CD4 count change (Figure 1B and Table 1). We also did not observe any significant association between CD4+ and CD8+ T-cell proliferation to anti-CD3 and SEB with CD4 count change (Table 1).

**Table 1:** Correlation between baseline immunological parameters and CD4 count change from baseline to nadir during TI

**T-cell compartment**	Proliferative Stimulus/Phenotypic Markers Expression*	Correlation with CD4 count change from baseline to nadir during TI**
**CD4**	anti-CD3	-0.07 (p = 0.87)
	SEB	0.43 (p = 0.30)
	CMV	-0.59 (p = 0.045)***
	Gag p55 peptide pool	-0.40 (p = 0.17)
	%CD57+	-0.69 (p = 0.0095)***
	CD57+ MFI	-0.03 (p = 0.93)
	%CD28	-0.38 (p = 0.19)
	CD28+ MFI	-0.18 (p = 0.55)
**CD8**	anti-CD3	-0.09 (p = 0.81)
	SEB	0.14 (p = 0.75)
	CMV	-0.48 (p = 0.12)
	Gag p55 peptide pool	-0.01 (p = 0.96)
	%CD57+	0.01 (p = 0.97)
	CD57+ MFI	0.29 (p = 0.34)
	%CD28	0.33 (p = 0.27)
	CD28+ MFI	-0.10 (p = 0.73)
Baseline CD4+ count		-0.61 (p = 0.030)***
Baseline viral load		-0.35 (p = 0.24)

*CMV = cytomegalovirus lysate; SEB = streptococcus enterotoxin B; MFI = mean fluorescence intensity.

**Spearman nonparametric correlation test (p-value).

***p < 0.05

**Figure 2:**
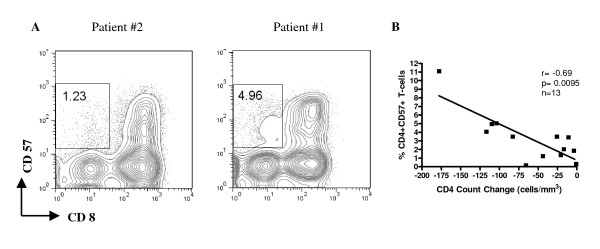
**Panel A shows FACS contour plots generated by staining PBMC from baseline time points from 2 study subjects.** Shown are CD3+ lymphocytes staining positively for CD57 but negatively for CD8, i.e. CD4+CD57+ cells. The number in the box in each plot indicates the percentage of CD4+CD57+ lymphocytes. Patient #2 on the left had a CD4 T-cell change during TI that was below the median while patient #1 on the right had a CD4 T-cell change that was above the median for the group. Panel B shows the correlation between the percentage of CD4+CD57+ cells at baseline and CD4 count loss during TI for all 13 patients tested. Spearman nonparametric correlation tests were used to assess the significance of the association between these parameters; *p *values <0.05 were considered significant.

Figure 2A shows flow cytometry contour plots for PBMC from 2 patients stained for CD57 on CD3+CD8- cells, which identify CD4+ T-cells. When results from all 13 patients were considered, we observed a negative correlation between the percent of CD4+CD57+ cells at baseline and CD4+ change during TI (r = -0.69, p = 0.0095; Spearman) (Figure 2B). All other phenotypic markers tested including expression of CD57 on CD8+ T-cells and CD28 expression on CD4+ and CD8+ T-cells did not correlate with CD4 count change during TI. (Table 1).

Patients were dichotomized into 2 groups by the median CD4 count change (-44 cells/mm^3^) and pre-TI immune parameters in the 2 subgroups were compared as another way of questioning whether ability to proliferate to antigens in the stimulatory panel or expression of CD57 or CD28 on T-cells were differentially distributed in the subgroups with lesser versus greater CD4 decline during TI. The group with a lesser CD4 count decline during TI (good responders) had a median change of -17 cells/mm^3 ^(IQR, -7, -23) whereas the group with the greater CD4 loss (poor responders) had a median change of -107 cells/mm^3 ^(IQR, -88, -115). Both good and poor responders had similar HIV-specific Gag p55 proliferative response {0% (IQR, 0, 0) in the CD4+ compartment and 0.07% (IQR, 0.03, 0.78) in the CD8+ compartment versus 0.11% (IQR, 0, 0.42) in the CD4+ compartment and 0.12% (IQR, 0.02, 0.25) in the CD8+ compartment for good and poor responders; p = 0.22, p = 0.72 respectively; Mann-Whitney test} (not shown). A non significant trend was observed for the subpopulation with the lesser CD4+ T-cell loss to have a lower percentage of CD4+CD57+ T-cells at baseline compared with the group with higher CD4+ T-cell decline {1.88% (IQR, 1.30, 2.74) versus 4.52% (IQR, 3.65, 5.04), p = 0.07; Mann-Whitney test}.

## Discussion

In this retrospective study, we showed that baseline CMV-specific CD4+ T-cell proliferation, pre-TI CD4 count, and percent CD4+CD57+ T-cells correlated negatively with CD4+ count change during a TI in patients experiencing virologic failure. Despite the known inverse association between plasma viremia and HIV-specific T-lymphocyte proliferative capacity, we found no association between the presence of HIV-specific proliferative responses at baseline and the extent of decline in CD4+ T-cells numbers during TI. In contrast, baseline CD4+ T-cell proliferation to CMV was an indicator of a larger decline in CD4 count during TI. Since more than 95% of HIV-infected patients are also infected with CMV, the presence of the endogenous CMV has the potential to activate and expand CD4+ T-cells, rendering them permissive for HIV replication and susceptible to virus mediated destruction [12]. Further research is needed to determine whether this mechanism underlies the association between CMV-specific proliferation and CD4+ T-cell decline during TI. One way to test our theory would be to examine whether there was a correlation between expression of immune activation markers such as CD38 and HLA-DR and proliferation to CMV. Unfortunately limitations in availability of cell numbers for these samples precluded including anti-CD38 or anti-DR in our antibody panels. However, we do recognize the importance of testing for CD38 and HLA-DR and we recommend the investigation of these activation markers in any future studies. Our findings differ from those of previous studies, in that baseline lymphoproliferative response to recall antigens in the setting of a TI initiated in early stage chronic HIV infection was a predictor of favorable virologic outcome following re-initiation of cART [13]. The discrepancies observed could be explained by the difference in the study population, disease stage, and endpoints measured.

Although pre-TI CD4 count nadir was not predictive of CD4+ T-cell decline as shown in previous studies [14,15], we observed that the pre-TI baseline CD4 count correlated negatively with CD4+ T-cell decline during TI. The use of CD4 count as a marker of clinical disease progression is well established in HIV infection. In advanced HIV infection where the individuals experienced virologic failure to all three ARV drug classes, baseline CD4 count was found to be a strong predictor of short-term risk of AIDS and death [16]. Here, we further underline the potential use of CD4 count in the setting of TI before initiating ensuing therapy.

In addition to using CD4 counts and viral loads to evaluate clinical HIV disease progression, cell surface markers including CD28 and CD57 have also been investigated. Traditionally, CD28 has been defined as a senescence marker whose loss of expression by T-cells during HIV infection has been associated with disease progression [6]. Brenchley et al. demonstrated that the expression of CD57 rather than the loss of CD28 expression defined replicative senescence [8]. They also showed that HIV-specific CD8+ T-cells that expressed CD57 were more sensitive to activation-induced apoptosis. More recent data further characterized CD4+CD57+ T-cells in HIV-infection as proliferation-incompetent and associated with an increased rate of spontaneous and activation-induced apoptosis [9]. We examined the expression of both markers and found that the baseline percentage of CD4+CD57+ T-cells correlated negatively with CD4 count loss during TI. Moreover the subpopulation with a lesser CD4+ T-cell decline during TI had a lower median percentage of CD4+CD57+ T-cells, although the difference did not reach statistical significance when compared to the subpopulation experiencing greater CD4+ T-cell loss during TI. Exposure to high antigen levels due to virologic failure in these patients may drive the CD4+ T-cell population to express CD57, a marker of terminal differentiation [9,17]. This CD4+CD57+ T-cells population identified at baseline was potentially more susceptible to cell death and could account for the greater CD4 decline during the viral rebound see during TI.

Some of the limitations of this study include the small sample size and the time interval between the baseline time point at which immune parameters were tested the start of TI. One individual was tested 11.3 months from start of TI, 5 were tested between 3 and 6 months of TI initiation and the remainder were tested for immune parameters between 0.7 and 2.5 months from start of TI. While it is possible that the measurements do not truly represent the state of the immune system at the time treatment is stopped, we contend that these investigations are still relevant in the context of an exploratory retrospective study. The immune parameters found to be of significance should be further validated in a prospective study with larger sample size and a synchronized baseline pre-TI time point for every patient.

## Conclusion

Our study identified immune parameters, which may predict CD4+ T-cell decline during a TI in the setting of virologic failure. Results presented may have important clinical implications for patients deciding to interrupt ARV treatment. Further investigation with larger sample size will be needed to fully evaluate these immune predictors.

## Competing interests

The authors declare that they have no competing interests.

## Authors' contributions

KHH contributed to the study design, acquisition of data, analysis and interpretation of results, and drafting the manuscript. MRL contributed to the study design, analysis and interpretation of results and manuscript preparation. CMT contributed to study subject recruitment and follow up, study design and manuscript preparation. NFB supervised the experimental component of the work, contributed to the study design, analysis and interpretation of results and manuscript preparation.

## Pre-publication history

The pre-publication history for this paper can be accessed here:


